# Prevalence and trends in mono- and co-infection of COVID-19, influenza A/B, and respiratory syncytial virus, January 2018–June 2023

**DOI:** 10.3389/fpubh.2023.1297981

**Published:** 2023-12-11

**Authors:** Min Kyung Lee, David Alfego, Suzanne E. Dale

**Affiliations:** Labcorp, Burlington, NC, United States

**Keywords:** COVID-19, co-infections, influenza, RSV, triple-demic

## Abstract

**Objectives:**

This study aimed to determine the impact of the COVID-19 pandemic on the overall prevalence and co-infection rates for COVID-19, influenza A/B, and respiratory syncytial virus in a large national population.

**Methods:**

We conducted a retrospective review of 1,318,118 multi-component nucleic acid amplification tests for COVID-19, influenza A/B, and RSV performed at Labcorp^®^ sites from January 2018 to June 2023, comparing positivity rates and co-infection rates by age, sex, and seasonality.

**Results:**

In 2021–2023, 1,232 (0.10%) tested positive for COVID-19 and influenza A/B, 366 (0.03%) tested positive for COVID-19 and RSV, 874 (0.07%) tested for influenza A/B and RSV, and 13 (0.001%) tested positive for COVID-19, influenza A/B, and RSV. RSV positivity rates were particularly higher in Q2 and Q3 of 2021 and in Q3 of 2022. Higher influenza A positivity proportions were found in Q4 of 2021 and again in Q2 and Q4 of 2022. Influenza B positivity had been minimal since the start of the pandemic, with a slight increase observed in Q2 of 2023.

**Conclusion:**

Our findings highlight the need for adaptability in preparation for upper respiratory infection occurrences throughout the year as we adjust to the COVID-19 pandemic due to the observed changes in the seasonality of influenza and RSV. Our results highlight low co-infection rates and suggest heightened concerns for co-infections during peaks of COVID-19, influenza, and RSV, which may perhaps be reduced.

## Introduction

The COVID-19 pandemic and the accompanying restrictions have changed the patterns of other respiratory pathogens. While circulation trends for traditional respiratory pathogens including influenza and respiratory syncytial virus (RSV) have been studied early in the COVID-19 pandemic (1–5), trends in a large population prior to and during the pandemic, especially in the fall of 2022 when concerns for a tripledemic (concurrent COVID-19, influenza A/B, and RSV peaks) were widespread, have been limited. We investigate COVID-19, influenza A/B, and RSV infections detected at a national commercial laboratory from January 2018 to June 2023 to determine the impact of the COVID-19 pandemic on the overall prevalence and co-infection rates for influenza and RSV.

## Methods

A retrospective review of 1,318,118 multi-component nucleic acid amplification tests (NAAT) for COVID-19, influenza A/B, and RSV performed at Labcorp^®^ sites from January 2018 to June 2023 was conducted to assess the prevalence of single virus detection compared to co-infections. Aggregate data from multiple test types were used (Supplementary Tables S1, S2). A total of 68,865,574 COVID-19-only NAAT results were included to serve as a reference for general COVID-19 positivity rates.

Prevalence ratios (PRs) adjusted for age were calculated with Mantel–Haenszel tests utilizing the meta R package (v6.2–1). Chi-squared tests were used to compare the sex distribution for each infection status to the overall distribution for each age group in R (v4.2.2). Subjects with unknown age or sex were removed when calculating prevalence ratios and chi-squared tests. Statistical significance thresholds were set to less than 0.05.

## Results

**Figure 1:**
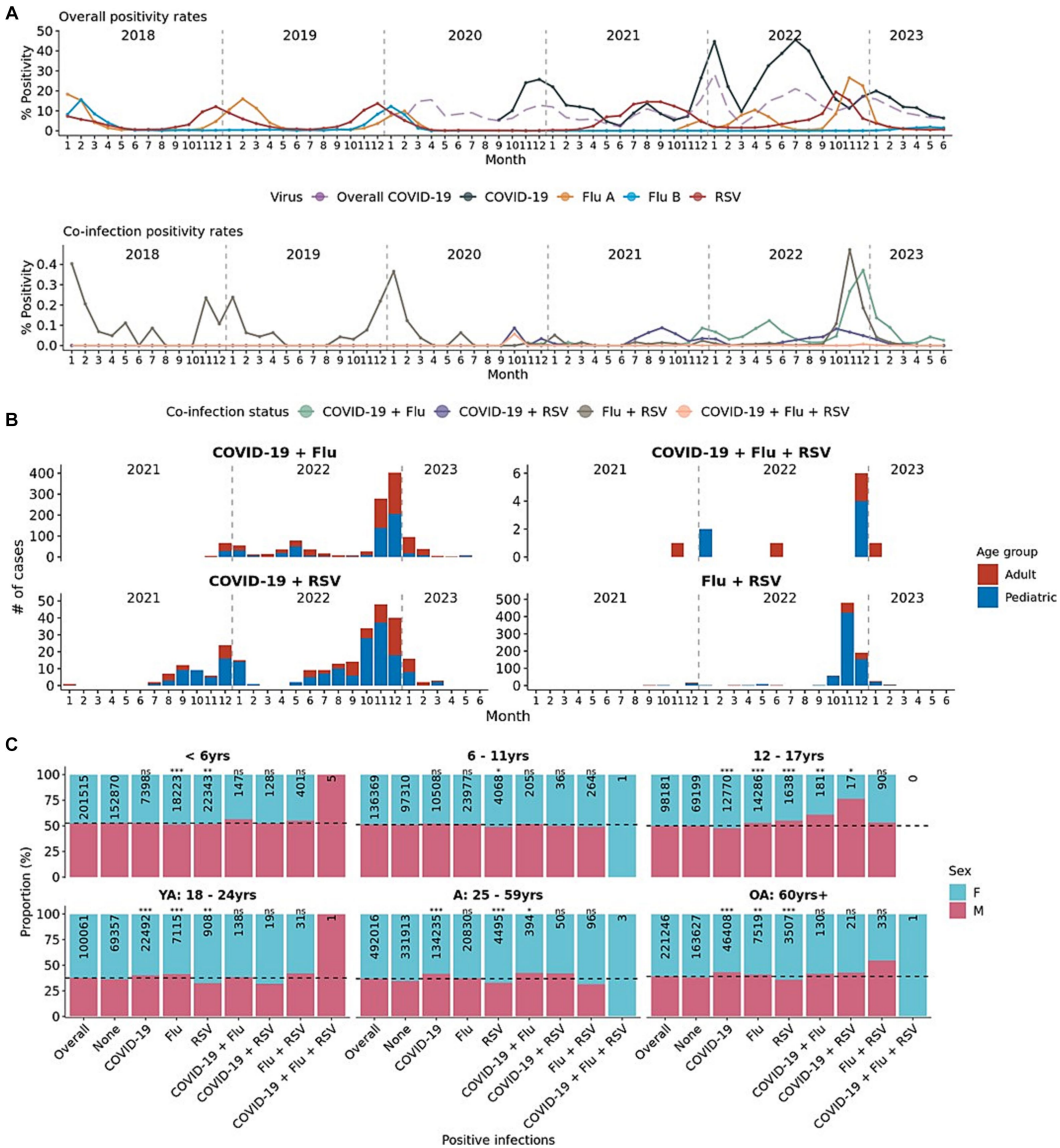
Mono- and co-infection prevalence of respiratory pathogens in January 2018–June 2023. **(A)** Overall and co-infection monthly positivity rates for COVID-19, influenza A, influenza B, and RSV. Long dashed purple lines indicate the positivity rates from 68 million+ COVID-19-only tests as a reference. **(B)** Number of co-infection cases over time from January 2021 to June 2023. Colored by the age group (pediatric and adult) of the patient. **(C)** Patient sex distribution by mono- or co-infection status for each age group. Distribution of sex for each positive infection is compared to the distribution of the overall tested population using the chi-squared test. *p*-value labels: *p < 0.05; **p < 0.01; ***p < 0.001; n.s. = not significant.

At the beginning of the COVID-19 pandemic, influenza A/B and RSV cases reduced in 2020 compared to 2019 (Figure 1A). Influenza and RSV began to re-emerge in 2021, but the seasonality of these viruses was different from the pre-pandemic years. While RSV typically peaks in early fall to winter (Q4), RSV positivity rates were particularly higher in the spring and summer of 2021 (prevalence ratio [95% confidence interval]: Q2: 4.59 [3.59–5.87]; Q3: 12.78 [9.39–17.39]) and in the late summer of 2022 (Q3 PR: 6.00 [4.40–8.19], Table 1). Although influenza A has historically peaked in early to mid-winter (Q1), higher influenza A positivity proportions were found in the later months of 2021 (Q4 PR: 1.41 [1.24–1.60]) and again in Q2 and Q4 of 2022 (PR: Q2: 2.61 [2.23–3.04]; Q4: 8.56 [7.57–9.68], Table 1). Influenza B positivity had been minimal since the start of the pandemic, with a slight increase observed in Q2 of 2023 (PR: 3.2 [2.19–4.69]; Figure 1A; Table 1). These respiratory pathogens generally did not peak in the same months, a pattern more evident when positivity rates were assessed by age (Supplementary Figure S1).

**Table 1:** Quarterly comparisons in positivity rates for each respiratory pathogen for each year to 2019 (age-adjusted prevalence ratio – 95% confidence interval).

	2023 vs 2019	2022 vs 2019	2021 vs 2019	2020 vs 2019
Flu A				
Q1	0.19 (0.18–0.2)	0.25 (0.23–0.26)	0 (0–0.01)	0.45 (0.43–0.48)
Q2	0.35 (0.29–0.43)	2.61 (2.23–3.04)	0.02 (0.01–0.04)	0.04 (0.02–0.08)
Q3		1.43 (0.97–2.1)	0.14 (0.08–0.24)	0.16 (0.05–0.5)
Q4		8.56 (7.57–9.68)	1.41 (1.24–1.6)	0.02 (0.01–0.04)
Flu B				
Q1	1.22 (0.91–1.65)	0.02 (0.01–0.04)	0.06 (0.02–0.16)	16.50 (12.35–22.04)
Q2	3.20 (2.19–4.69)	0.01 (0.00–0.02)	0.07 (0.02–0.18)	0.20 (0.08–0.51)
Q3		0.01 (0.01–0.03)	0.06 (0.03–0.12)	0.14 (0.04–0.52)
Q4		0.01 (0.01–0.01)	0.01 (0–0.01)	0.01 (0.01–0.03)
RSV				
Q1	0.45 (0.41–0.49)	0.3 (0.27–0.33)	0.1 (0.07–0.13)	0.79 (0.72–0.87)
Q2	0.50 (0.38–0.66)	1.74 (1.37–2.21)	4.59 (3.59–5.87)	0.32 (0.21–0.49)
Q3		6.00 (4.40–8.19)	12.78 (9.39–17.39)	0.06 (0.01–0.35)
Q4		1.45 (1.36–1.54)	0.92 (0.86–0.99)	0.01 (0.01–0.03)

**Figure 2:**
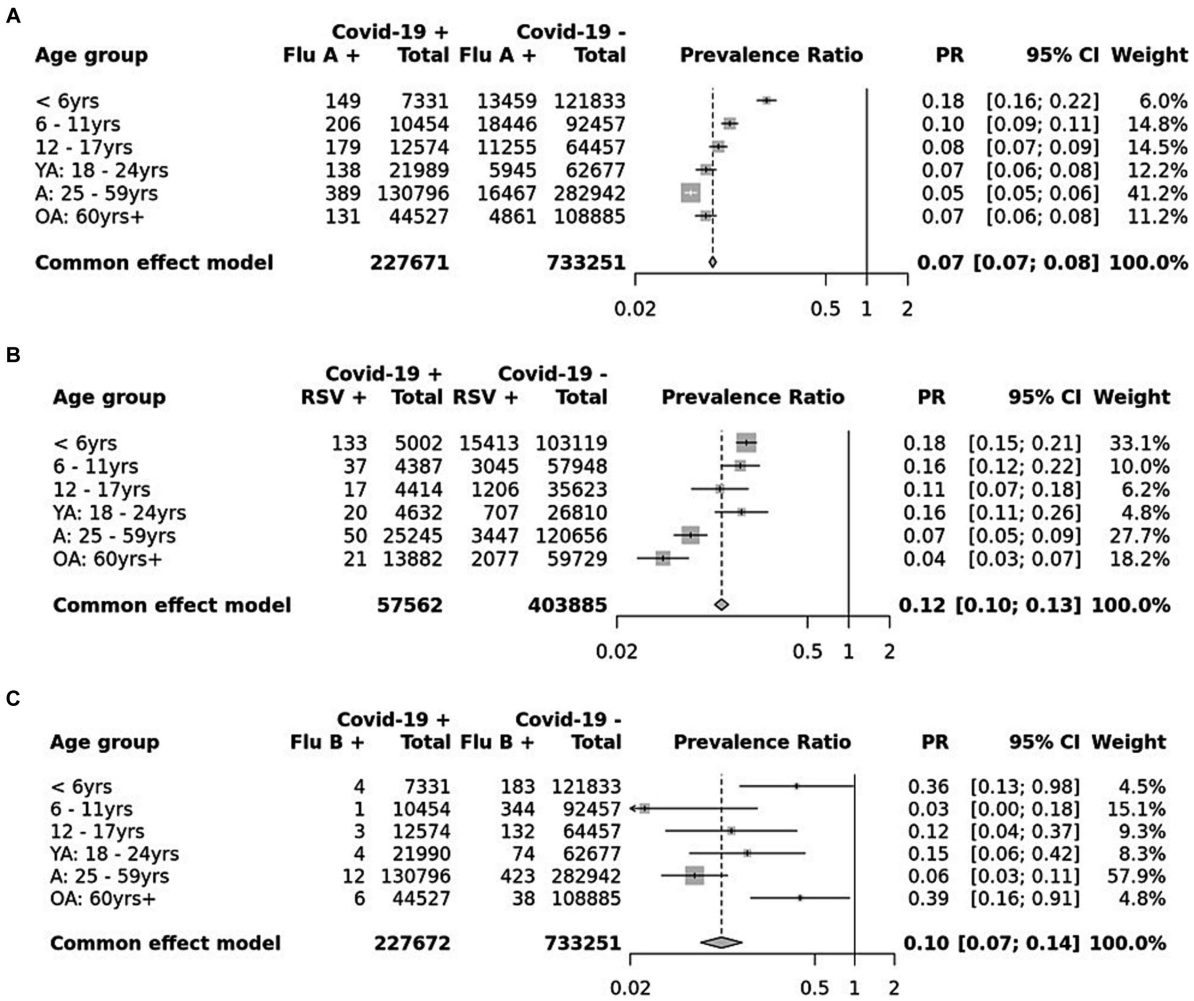
Age-adjusted prevalence ratio of those who tested positive for COVID-19 and co-infected with **(A)** influenza A, **(B)** influenza B, and **(C)** RSV. Adjusted for age with Mantel–Haenszel tests.

Among 1,177,020 patients from 2021 to 2023, 1,232 (0.10%) tested positive for COVID-19 and influenza A/B, 366 (0.03%) tested positive for COVID-19 and RSV, 874 (0.07%) tested positive for influenza A/B and RSV, and 13 (0.001%) tested positive for COVID-19, influenza A/B, and RSV (Figure 1B). 5 of 11 (55%) patients that tested positive for all three were male patients under 6 years of age (Figure 1C). The distribution of sex in any co-infection status (COVID-19 + Flu; COVID-19 + RSV; Flu + RSV) generally was not different from the expected distribution (Figure 1C). Only in the 12–17-year-olds were there more male patients co-infected with COVID-19 and influenza A/B or COVID-19 and RSV than expected. The proportion of COVID-19 subjects who were positive for influenza A (PR: 0.07 [0.07 - 0.08]), influenza B (PR: 0.10 [0.07 - 0.14]), or RSV (PR: 0.12 [0.10 - 0.13]) were lower than the proportion of COVID-19 positive subjects who were not co-infected with other respiratory viruses (Figures 2A–C). Although the prevalence ratios were very low for all influenza A, influenza B, and RSV, subjects under 6 years of age had the highest proportions of co-infection with COVID-19 out of all age groups (Figures 2A–C).

## Discussion

Our results demonstrate the changes in the seasonality of influenza and RSV throughout the COVID-19 pandemic compared to before the pandemic. By interrogating a dataset from a large national reference laboratory, we identified the frequency of co-infection rates with COVID-19 and other common respiratory viruses to be relatively low, suggesting that concerns about COVID-19 co-infections with other traditional respiratory infections during the periods of higher COVID-19, influenza, and RSV positivity rates may be reduced. However, heightened pressure in healthcare systems from increased hospitalizations from infections and staff shortages (6) during a tripledemic may still be of significant concern.

Due to potentially overlapping symptoms observed in patients with COVID-19 and traditional respiratory pathogens, co-testing can provide a specific diagnosis, which may have treatment implications. Our results may have been impacted by differences in testing practices between the pandemic and pre-pandemic years, when patients may have been diagnosed in the absence of test results. Our results may also have been affected by the exclusion of antigen tests, which increased in use throughout the pandemic.

## Data availability statement

The datasets presented in this article are not readily available because the datasets generated and/or analyzed during the current study are not publicly available due to proprietary information from Labcorp. Requests to access the datasets should be directed to leem62@labcorp.com.

## Ethics statement

Ethical approval was not required for the studies involving humans because completely de-identified results from commercial diagnostic testing were used in analysis. The studies were conducted in accordance with the local legislation and institutional requirements. The human samples used in this study were acquired from a by-product of routine care or industry. Written informed consent to participate in this study was not required from the participants or the participants’ legal guardians/next of kin in accordance with the national legislation and the institutional requirements.

## Author contributions

ML: Data curation, Formal analysis, Investigation, Methodology, Visualization, Writing – original draft, Writing – review & editing. DA: Conceptualization, Data curation, Project administration, Supervision, Writing – review & editing. SD: Conceptualization, Investigation, Project administration, Supervision, Writing – review & editing.
