# Supplementary material for: Prevalence and trends in mono- and co-infection of COVID-19, influenza A/B, and respiratory syncytial virus, January 2018–June 2023
Source: Front Public Health. 2023 Dec 11;11:1297981. doi: 10.3389/fpubh.2023.1297981 (PMC10754957; doi:10.3389/fpubh.2023.1297981)
Supplement: Supplementary file 1 [file Data_Sheet_1.docx]

**Supplementary Table 1. Respiratory pathogens included in each Labcorp test**

|  | **Labcorp  Test Number** | **SARS-CoV-2  (COVID-19)** | **Influenza A  (Flu A)** | **Influenza B  (Flu B)** | **Respiratory Syncytial Virus  (RSV)** | **# of tests (N = 1,318,118)** | **Years used** |
| --- | --- | --- | --- | --- | --- | --- | --- |
| Panel tests | **139250** |  | ✓ | ✓ | ✓ | 56,357 | 18, 19, 20 |
|  | **139650** |  | ✓ | ✓ | ✓ | 175,134 | 18, 19, 20, 21, 22, 23 |
|  | **140140** | ✓ | ✓ | ✓ | ✓ | 478,555 | 20, 21, 22, 23 |
|  | **140147** | ✓ | ✓ | ✓ |  | 534,910 | 20, 21, 22, 23 |
|  | **140163** |  | ✓ | ✓ | ✓ | 17,196 | 20, 21, 22, 23 |
|  | **140165** |  | ✓ | ✓ |  | 22,490 | 20, 21, 22, 23 |
|  | **140172** | ✓ |  |  | ✓ | 33,476 | 20, 21, 22, 23 |
| COVID-19 only test | **139900** | ✓ |  |  |  | 68,865,574 | 20, 21, 22, 23 |

**Supplementary Table 2. Distribution of age groups and sex in the subject population.**

| **Age group** |  |
| --- | --- |
| < 6yrs | 201974 (15.3%) |
| 6 - 11yrs | 136610 (10.4%) |
| 12 - 17yrs | 98324 (7.5%) |
| YA: 18 - 24yrs | 100397 (7.6%) |
| A: 25 - 59yrs | 493561 (37.4%) |
| OA: 60yrs+ | 222177 (16.9%) |
| UNK | 65075 (4.9%) |
| **Sex** |  |
| F | 750156 (56.9%) |
| M | 566313 (43.0%) |
| U | 1649 (0.1%) |
