# Supplementary figures and images for: Prevalence and trends in mono- and co-infection of COVID-19, influenza A/B, and respiratory syncytial virus, January 2018–June 2023
Source: Front Public Health. 2023 Dec 11;11:1297981. doi: 10.3389/fpubh.2023.1297981 (PMC10754957; doi:10.3389/fpubh.2023.1297981)

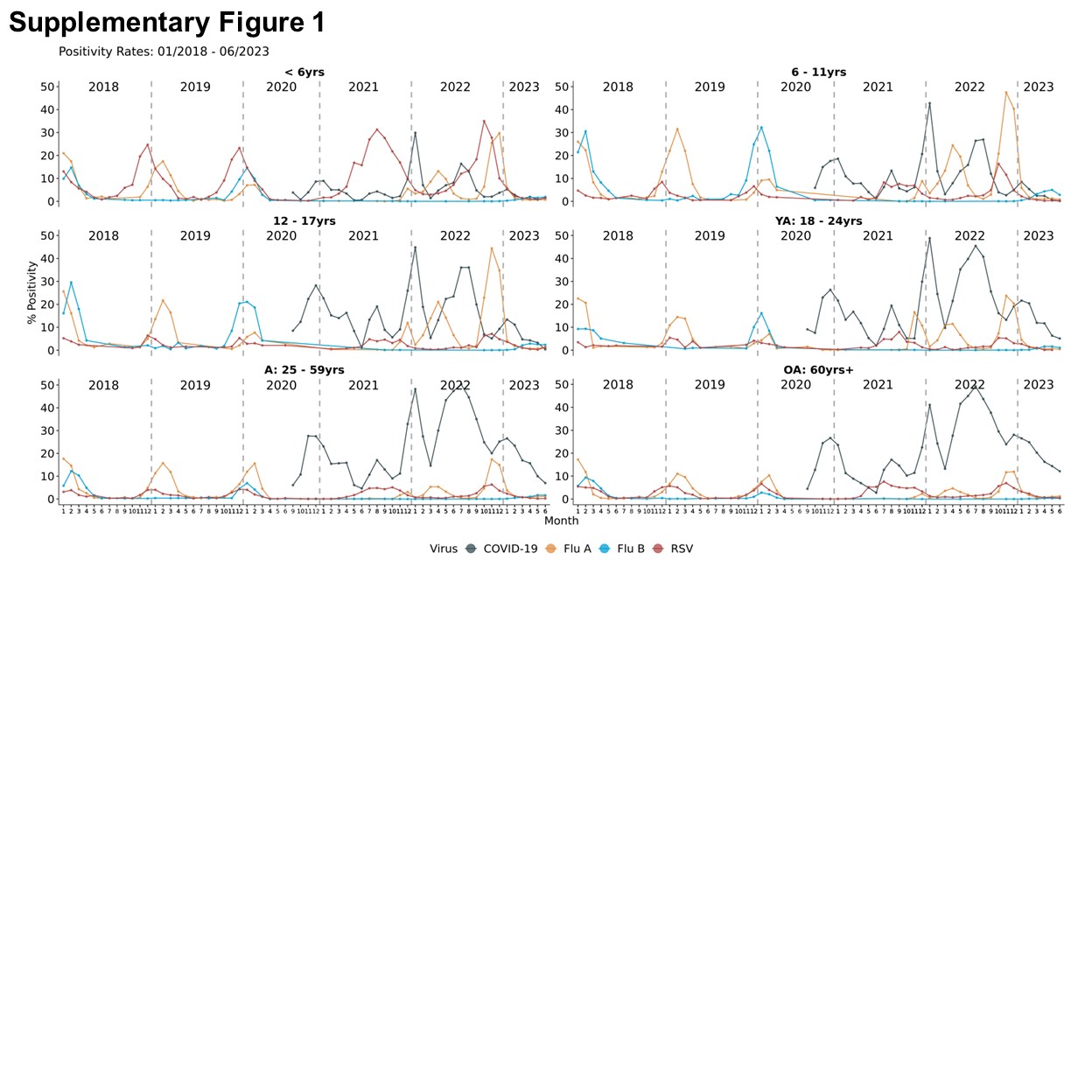

Supplement: Supplementary file 2 [file Image_1.JPEG]
